# Eye movement desensitization and reprocessing in trauma related panic disorder: clinical outcomes through a neuroendocrine lens

**DOI:** 10.1097/MD.0000000000050943

**Published:** 2026-10-16

**Authors:** Emrah Yildiz, Zeynel Abidin Sayiner

**Affiliations:** a Department of Psychiatry, Private Clinic, Gaziantep, Turkey; b Department of Endocrinology and Metabolism, Gaziantep SANKO University School of Medicine, Gaziantep, Turkey.

**Keywords:** Eye Movement Desensitization Reprocessing, panic disorder, psychological trauma, psychotherapy

## Abstract

**Background:**

This study aimed to evaluate the effect of Eye Movement Desensitization and Reprocessing (EMDR) therapy on symptoms and disease severity in individuals diagnosed with panic disorders who had experienced at least 1 traumatic event.

**Methods:**

This study was conducted using a single-group pretest posttest experimental design. Participants received the standard EMDR protocol, and symptom severity was assessed using the Panic Disorder Severity Scale (PDSS) at 4 separate time points (pretreatment, post-first session, post-second session, and posttreatment).

**Results:**

The study included 34 participants (25 women, 9 men; mean age, 33.6 ± 8.1 years). A significant decrease in PBSS scores was observed across the 4 measurements (pretest: 19.6 ± 5.7; posttest: 8.8 ± 2.7; *P* < .001). The change (delta) in PBSS scores according to sex was 11.5 ± 3.9 in women and 9.1 ± 2.6 in men, with no statistically significant difference between groups (*P* = .068). No effect of marital status on symptom changes was observed (*P* > .05). A weak but nonsignificant positive correlation was found between age and PBSS change scores (*r* = 0.261; *P* = .135).

**Conclusion:**

EMDR therapy appears to be effective in reducing symptom severity in patients with panic disorders in the short term. This effect was observed independently of the demographic variables. However, further studies are needed to determine long term efficacy and compare the results with other therapeutic approaches.

## 1. Introduction

Trauma is the result of an individual being exposed to threatening or frightening events. Individuals experience emotional responses, including fear and horror, as a result of trauma. Traumatic experiences are those that disrupt an individual’s sense of normalcy and daily routine.^[1]^ Abuse, accidents, natural disasters, rape, violence, and assault are examples of traumatic experiences. Events that are not part of the typical course of daily life are fundamental components of traumatic experiences. Panic disorder (PD) may arise from traumatic experiences.^[2]^ PD is characterized by abrupt panic attacks without specific circumstances or triggers. Panic attacks encompass physical symptoms such as fear, palpitations, elevated heart rate, perspiration, dyspnea, dizziness, trembling, and hot flashes, accompanied by a profound sense of fear. Panic disorder, characterized by recurrent panic attacks, can profoundly affect an individual’s professional, familial, social, and educational spheres.^[3]^ The diagnostic criteria for panic attacks, designated as the onset of PD in the fifth edition (Diagnostic and Statistical Manual of Mental Disorders, Fifth Edition [DSM-5]) of the American Psychiatric Association’s (APA) manual for diagnosing and classifying mental disorders, are as follows: chest tightness, dizziness, palpitations, sweating, a sense of choking, chills or hot flashes, fear of dying, numbness or tingling in the body, and fear of losing control.^[4]^ This condition may manifest abruptly during periods of tranquility or anxiety. Panic disorder is diagnosed when 4 or more of these symptoms are present.

EMDR Therapy is a psychotherapy approach that posits that repetitive eye movements can alleviate distressing thoughts. Francine Shapiro developed it in 1987. EMDR Therapy comprises 8 stages: history-taking, preparation, assessment, desensitization, installation, body scan, and closure. The initial step in the process is to evaluate the patient’s dysfunctional thoughts and beliefs in relation to the event they have experienced, after assessing their readiness and taking their history. The primary objective of this treatment method is to assist patients in regaining control over their distressing thoughts.^[5]^ In the literature, medication and cognitive behavioral interventions are the primary treatments for PD when examining meta-analysis studies.^[6]^ Medication regulates the impaired neurotransmitter system in the brain. Cognitive-behavioral interventions encompass homework assignments, relaxation, exposure, and instruction of skills to mitigate anxiety intensity, such as breathing exercises. Research on the treatment of PD reveals that both medication and cognitive behavioral therapy are successful.^[7,8]^ Additionally, PD is mediated by the hypothalamic–pituitary–adrenal (HPA) axis, leading to cortisol, catecholamines, and related autonomic dysregulation. Chronic activation or sensitization of this axis, often triggered by unresolved trauma, can lead to persistent neuroendocrine imbalances that exacerbate both physiological and psychological symptoms. EMDR may indirectly support the reorganization of the HPA axis by facilitating the adaptive processing of traumatic memories, thereby reducing the reactivity of stress hormones and contributing to improvements in panic symptoms.^[9,10]^ This study aimed to evaluate the effect of EMDR therapy on symptom severity in patients with panic disorders with traumatic experiences. Additionally, the regulatory potential of EMDR on cognitive and physiological stress responses was examined, and its effectiveness as an alternative or complementary method for treating PD was investigated.

## 2. Materials and methods

### 2.1. Participants

This study was an experimental single-group pretest and posttest intervention study. The study group consisted of individuals diagnosed with PD based on the DSM-5 criteria who had experienced at least 1 traumatic event. Participants were selected from patients who voluntarily applied for treatment. The inclusion criteria were as follows: age between 18 and 60 years, diagnosis of PD, and reporting at least 1 traumatic experience. The exclusion criteria were a diagnosis of psychotic disorder, active substance use, and severe cognitive impairment. The study protocol was approved by the Gaziantep University Clinical Research Ethics Committee (Approval No. 2023/103). All participants provided written informed consent before participation.

### 2.2. Forms administered to participants

#### 2.2.1. Socio-Demographic Information Form

A structured form containing information such as participants’ age, gender, marital status, number of siblings, educational status, psychiatric illness history, psychiatric history in parents, exposure to traumatic experiences, and timing of traumatic events.

#### 2.2.2. Panic Disorder Severity Scale (PDSS)

The purpose was to determine the severity of panic disorder symptoms. The scale was administered to participants at 4 different time points: before therapy (pretest), after the first EMDR session, after the second EMDR session, and after the final session.

### 2.3. Statistical analysis

Statistical analyses were conducted using the Statistical Package for the Social Sciences (SPSS) version 25.0 software. Categorical variables were summarized as counts and percentages, whereas continuous variables were summarized as means and standard deviations (with medians and ranges provided when necessary). The Kolmogorov–Smirnov test was employed to ascertain whether the parameters in the study exhibited a normal distribution. The Mann–Whitney *U* test was used for parameters exhibiting a non-normal distribution. Spearman rho correlation test was employed to analyze the disparities among continuous measurement parameters. The significance level for all tests was set at .05.

## 3. Results

The study included 34 patients, of whom 25 (73.5 %) were female and 9 (26.5%) were male. The mean age of the patients was 33.6 ± 8.1 years (median: 33). Fifteen (44.1%) patients were single and 19 (55.9%) were married. The baseline demographic characteristics of the participants are presented in Table 1.

**Table 1:** Demographic characteristics of the participants.

	Count	Percentage
Age (year ± SD)	33.6 ± 8.1	33 (18–54)
Gender (female/male)	25/9	73.5/26.5
Marital status (single/married)	15/19	44.1/55.9

SD = standard deviation.

The PDSS scores administered to patients at 4 different time points were 19.6 ± 5.7 at the first measurement, 16.3 ± 4.7 at the second measurement, 12.6 ± 3.7 at the third measurement, and 8.8 ± 2.7 at the fourth measurement. The changes in the PDSS scores administered to patients at 4 different time points were statistically significant (*P* < .001). The PDSS score obtained at the fourth time point was significantly lower than the PDSS scale score at the first measurement (*P* < .001). A significant reduction in PDSS scores across the 4 assessment points is shown in Table 2.

**Table 2:** Changes in Panic Disorder Severity Scale (PDSS) scores across 4 assessment points.

	First	Second	Third	Fourth	*P*	*P*
PDSS mean score	19.6 ± 5.7	16.3 ± 4.7	12.6 ± 3.7	8.8 ± 2.7	<.001*	<.001*

p1: Change between the first, second, and fourth measurements, p2: Difference between the first and fourth measurements, Wilcoxon sign rank test.

PDSS = Panic Disorder Severity Scale.

* *P* < .01.

No statistically significant difference was observed in the PDSS scores between female and male patients (*P* > .05). No significant difference was noted between the groups regarding delta scores, reflecting the disparity between the initial and final measurements (11.5 ± 3.9 in women; 9.1 ± 2.6 in men; *P* = .068). Table 3 illustrates the comparison of PDSS scores between female and male participants at each measurement time point.

**Table 3:** Comparison of PDSS scores between genders across all assessment points.

	Female (mean ± SD)	Male (mean ± SD)	*P*
First	20.4 ± 5.9	17.6 ± 5.0	.132
Second	16.9 ± 4.9	14.7 ± 3.9	.188
Third	12.8 ± 3.8	11.9 ± 3.3	.556
Fourth	8.9 ± 2.6	8.4 ± 3.0	.937
Delta (∆)	11.5 ± 3.9	9.1 ± 2.6	.068

Delta (∆): Difference between the first measurement PDSS and the fourth measurement PDSS.

PDSS = Panic Disorder Severity Scale, SD = standard deviation.

No significant difference was noted between the PDSS scores and the marital status variable. The comparison of PDSS scores based on marital status is summarized in Table 4 (*P* > .05).

**Table 4:** Comparison of PDSS scores according to marital status.

	Single (mean ± SD)	Married (mean ± SD)	*P*
First	18.9 ± 5.5	20.4 ± 5.9	.289
Second	15.2 ± 4.4	17.2 ± 4.8	.133
Third	12.1 ± 3.6	12.9 ± 3.8	.403
Fourth	8.3 ± 2.6	9.1 ± 2.8	.275
Delta (∆)	10.3 ± 3.9	11.3 ± 3.7	.337

Delta (∆): difference between the first PDSS measurement and the fourth PDSS measurement.

PDSS = Panic Disorder Severity Scale, SD = standard deviation.

A low positive correlation was observed between the PDSS scores obtained in the first measurement and age (*r* = 0.247); however, this correlation was not statistically significant (*P* = .159). Similarly, in the second measurement, the correlation coefficient was *r* = 0.285, and the significance level was *P* = .102. In the third measurement, the relationship remained weak (*r* = 0.145), and the *P*-value was .414. In the fourth measurement, *r* = 0.263 and *P* = .133; the delta (difference between the first and fourth measurements) between PDSS scores and age was *r* = 0.261 and *P* = .135. These results indicate that age does not have a significant effect on changes in symptom severity during the EMDR therapy process (*P* > .05; Table 5).

**Table 5:** Correlation between PDSS scores and age at each assessment point.

	Age	
	*r*	*P*
First	0.247	.159
Second	0.285	.102
Third	0.145	.414
Fourth	0.263	.133
Delta (∆)	0.261	.135

Spearman rho correlation, *r* = correlation coefficient, Delta (∆): Difference between the first measurement PDSS and the fourth measurement.

PDSS = Panic Disorder Severity Scale.

## 4. Discussion

EMDR application demonstrated a statistically significant decrease in PDSS scores over 4 sessions. Our findings are consistent with those of a comprehensive analysis conducted by Faretta and Dal Farra, who reviewed 6 randomized controlled trials.^[11]^ This review highlighted that EMDR therapy has positive effects on panic disorder and phobias, particularly in terms of significant reductions in anxiety and avoidance behaviors. However, 1 study reported partial results, while another reported an ineffective outcome. These differences can be attributed to factors such as protocol adherence, session duration, and therapist’s experience. In a comparative study conducted by Faretta, EMDR and cognitive-behavioral therapy were compared in the treatment of panic disorder, and both therapies were found to be effective in reducing symptoms.^[12]^ However, EMDR was reported to provide symptom improvement in a shorter time, and this effect was maintained in follow-up measurements. Similarly, a randomized controlled study by Horst et al demonstrated that EMDR therapy and CBT have equivalent efficacy in the treatment of panic disorder. In particular, the frequency of panic attacks was lower in individuals who underwent EMDR than in those who underwent CBT.^[13]^ In a case study presented by Bhagwagar, panic attacks completely disappeared, and treatment gains were maintained for 5 years following a 17-session treatment in which early childhood traumas were processed using EMDR. This long-term follow-up demonstrates that EMDR provides not only symptom suppression but also deep healing by enabling comprehensive processing of traumatic memories.^[14]^ On the other hand, another study reported that EMDR was effective in some measures when compared to placebo and waiting list control groups but did not produce a significant difference in areas such as panic attack frequency and anxious thoughts. However, the study noted statistical limitations, such as low sample power, which may have obscured the true effect of EMDR.^[12]^ The findings of our study support the strong therapeutic effect of EMDR in panic disorder developed because of trauma. The reduction in symptoms was observed at both cognitive and physical levels, which can be explained by EMDR’s adaptive information processing model.^[15]^

EMDR enables individuals to reprocess unprocessed traumatic memories, transforming the automatic thoughts and physiological responses associated with these memories.^[16]^ This can reduce the frequency and severity of panic attacks and improve cognitive and emotional functioning. The absence of a significant relationship between symptom severity and demographic variables such as gender, age, and marital status in the study may indicate that EMDR can be effective in a wide range of patient groups, regardless of such characteristics. From a pathophysiological perspective, EMDR therapy can reduce the sensory and emotional load of traumatic memories by reprocessing them, thereby reducing the pressure on the HPA axis and normalizing the chronic stress response. Some studies have reported findings such as reduced cortisol release, normalization of cortisol/ACTH ratios, and restoration of homeostasis following EMDR therapy.^[17]^ Epigenetic studies suggest that methylation changes in genes associated with the HPA axis are observed in panic disorder and that these changes can be modulated by psychotherapeutic interventions such as EMDR.^[18]^ These findings indicate that EMDR may have restorative effects not only at the psychological level but also at the biological level. However, the limited sample size requires that these relationships be retested in larger populations to generalize the results of our study.

EMDR therapy may provide a meaningful short-term effect in reducing symptom severity in patients with panic disorder with traumatic experiences. EMDR may be an effective intervention method for treating panic disorder. However, randomized controlled trials with larger sample sizes are needed to evaluate whether its effectiveness is sustained in the long term.

## Author contributions

**Conceptualization:** Emrah Yildiz, Zeynel Abidin Sayiner.

**Data curation:** Emrah Yildiz.

**Formal analysis:** Emrah Yildiz.

**Funding acquisition:** Emrah Yildiz.

**Investigation:** Emrah Yildiz.

**Methodology:** Emrah Yildiz.

**Project administration:** Emrah Yildiz.

**Resources:** Emrah Yildiz.

**Software:** Emrah Yildiz.

**Supervision:** Emrah Yildiz, Zeynel Abidin Sayiner.

**Validation:** Emrah Yildiz.

**Visualization:** Emrah Yildiz.

**Writing – original draft:** Emrah Yildiz, Zeynel Abidin Sayiner.

**Writing – review & editing:** Emrah Yildiz, Zeynel Abidin Sayiner.

## References

[1] Jovanovic T Ressler KJ. How the neurocircuitry and genetics of fear inhibition may inform our understanding of PTSD. Am J Psychiatry. 2010;167:648–62. 10.1176/appi.ajp.2009.09071074 PMC3603297 20231322

[2] Pearce L. Supporting children after a traumatic event. Nurs Standard. 2017;32:15. 10.7748/ns.32.1.15.s16 28853375

[3] Collison JR Correll TL. Treating panic disorder: a psychotherapeutic case. Innov Clin Neurosci. 2011;8:33–40. PMC3071090 21468296

[4] Hussain M Shah IA. Symptoms of panic disorder may lead to a potential misdiagnosis as a cardiopulmonary disease, particularly in rural areas. J Pak Med Assoc. 2024;74:2219. 10.47391/JPMA.21108 39659008

[5] Shapiro F. Eye Movement desensitization and reprocessing (EMDR): evaluation of controlled PTSD research. J Behav Ther Exp Psychiatry. 1996;27:209–18. 10.1016/s0005-7916(96)00029-8 8959422

[6] Cuijpers P Gentili C Banos RM Garcia-Campayo J Botella C Cristea IA. Relative effects of cognitive and behavioral therapies on generalized anxiety disorder, social anxiety disorder and panic disorder: a meta-analysis. J Anxiety Disord. 2016;43:79–89. 10.1016/j.janxdis.2016.09.003 27637075

[7] Pompoli A Furukawa TA Efthimiou O Imai H Tajika A Salanti G. Dismantling cognitive-behaviour therapy for panic disorder: a systematic review and component network meta-analysis. Psychol Med. 2018;48:1945–53. 10.1017/S0033291717003919 PMC6137372 29368665

[8] Barrera T Grubbs KM Kunik ME Teng EJ. A review of cognitive behavioral therapy for panic disorder in patients with chronic obstructive pulmonary disease: The rationale for interoceptive exposure. J Clin Psychol Med Settings. 2014;21:144–54. 10.1007/s10880-014-9393-4 24699909

[9] Tafet GE Nemeroff CB. Pharmacological treatment of anxiety disorders: the role of the HPA axis. Front Psychiatry. 2020;11:443. 10.3389/fpsyt.2020.00443 PMC7243209 32499732

[10] Flandreau EI Ressler KJ Owens MJ Nemeroff CB. Chronic overexpression of corticotropin-releasing factor from the central amygdala produces HPA axis hyperactivity and behavioral anxiety associated with gene-expression changes in the hippocampus and paraventricular nucleus of the hypothalamus. Psychoneuroendocrinology. 2012;37:27–38. 10.1016/j.psyneuen.2011.04.014 PMC3164918 21616602

[11] Faretta E Dal Farra M. Efficacy of EMDR therapy for anxiety disorders. J EMDR Pract Res. 2019;13:325–32.

[12] Goldstein AJ de Beurs E Chambless DL Wilson KA. EMDR for panic disorder with agoraphobia: comparison with waiting list and credible attention-placebo control conditions. J Consult Clin Psychol. 2000;68:947–56. 11142547

[13] Horst F Den Oudsten B Zijlstra W de Jongh A Lobbestael J De Vries J. Cognitive behavioral therapy vs. Eye movement desensitization and reprocessing for treating panic disorder: a randomized controlled trial. Front Psychol. 2017;8:1409. 10.3389/fpsyg.2017.01409 PMC5563354 28868042

[14] Bhagwagar H. EMDR in the treatment of panic disorder with agoraphobia: a case description. J EMDR Pract Res. 2016;10:256–74.

[15] Velu ME Kuiper RM Schok M Sleijpen M de Roos C Mooren T. Effectiveness of trauma-focused treatments for refugee children: a systematic review and meta-analyses. Eur J Psychotraumatol. 2025;16:2494362. 10.1080/20008066.2025.2494362 PMC12090257 40387621

[16] Inci Izmir SB Korkmazlar U Ercan ES. Eye movement desensitization and reprocessing therapy in adolescents with panic disorder: a twelve-week follow-up study. Clin Child Psychol Psychiatry. 2024;29:966–81. 10.1177/13591045231184757 37328192

[17] Usta M Gumus YY Say GN Bozkurt A Şahin B Karabekiroğlu K. Basal blood DHEA-S/cortisol levels predicts EMDR treatment response in adolescents with PTSD. Nord J Psychiatry. 2018;72:164–72. 10.1080/08039488.2017.1406984 29171317

[18] Zou Z Xiang M Zhang Y. Associations of DNA methylation of HPA axis-related genes and neuroendocrine abnormalities in panic disorder. Psychoneuroendocrinology. 2022;142:105777. 10.1016/j.psyneuen.2022.105777 35504198

